# Case Report: Spontaneous Appendicitis With Suspected Involvement of *Klebsiella variicola* in Two Pet Rabbits

**DOI:** 10.3389/fvets.2021.779517

**Published:** 2021-12-08

**Authors:** Vladimir Jekl, Anna Piskovska, Ivana Drnkova, Misa Skoric, Karel Hauptman, Jan Chloupek

**Affiliations:** ^1^Department of Pharmacology and Pharmacy, Faculty of Veterinary Medicine, University of Veterinary Sciences Brno, Brno, Czechia; ^2^Jekl & Hauptman Veterinary Clinic, Brno, Czechia; ^3^Department of Pathological Morphology, Faculty of Veterinary Medicine, Veterinary University, Brno, Czechia

**Keywords:** rabbit, appendix, appendicitis, gastrointestinal stasis, acute abdomen, *Klebsiella*

## Abstract

Although laboratory rabbits are commonly used as models of appendicitis in man, spontaneous appendicitis was only described *ante-mortem* in one pet rabbit with an acute abdomen. The aim of this article is to describe two spontaneous cases of appendicitis in pet rabbits, to describe therapeutic appendectomy, and to discuss the microbial flora of the inflamed appendix. A 5-month-old intact female and a 16-month-old, neutered male were presented to the veterinary clinic with restlessness, anorexia, and reduced faecal output. The main clinical findings were restlessness, severe discomfort on abdominal palpation, a mid-abdominal palpable tubulous mass and an elevated rectal temperature. Blood analyses showed lymphocytosis, monocytosis, and hyperglycaemia. Radiography was inconclusive. Abdominal ultrasound revealed a presence of a tubular structure with wall thicknesses of 4.2 and 3.7 mm in the two rabbits, respectively. The tubular structure had a rounded, closed end, and a multilayered wall, suggestive of appendicitis. Due to metabolic acidosis and poor prognosis, the first rabbit was euthanized. In the 16-month-old rabbit, appendectomy was performed. Recovery was uneventful, and 4 h after surgery, the rabbit started to become normally active. Postoperative care consisted of fluid therapy, multimodal analgesia, supportive care and prokinetics. Follow-up examinations at 10 days, 1 month, and at 11 months after the surgery did not show any abnormal clinical or laboratory findings. Histopathological examination of appendices from both rabbits showed gangrenous appendicitis. Aerobic cultivation showed the presence of pure culture of *Klebsiella variicola* sensitive to enrofloxacin, marbofloxacin, tetracycline, cefuroxime, trimethoprim sulphonamide, neomycin, and gentamicin. Restlessness associated with anorexia, abdominal pain, palpable abdominal mass, hyperglycaemia, lymphocytosis, and elevated rectal temperature may be indicative of inflammation within the gastrointestinal tract. Abdominal ultrasound is recommended in rabbits with showing these clinical signs because radiography can be inconclusive. Appendicitis is a life-threatening condition, which should be included into the list of differential diagnoses; for the rabbit, an acute abdomen and gastrointestinal stasis syndrome and must be treated immediately. *K. variicola* may be associated with appendicitis in rabbits as a causative agent or in association with appendix intraluminal dysmicrobia.

## Introduction

The vermiform appendix (*processus vermiformis caeci*) of the rabbit is the blind end of the caecum, 9–12 cm in length, ending on the left flank, dorsal to the first segment of the caecum (1–3). In the rabbit, the appendix is an important site for early development and diversification of the B-cell repertoire and is one of the major parts of the gut-associated lymphoid tissues (1, 4). On ultrasound examination, the appendix can be visualised through the median and/or left paramedian views as a tubular structure with a rounded, closed end and a multilayered wall characteristic of the intestinal tract. The normal appendix wall thickness measured by ultrasound is 1.1–2.1 mm, and the normal appendix diameter is 3.9–8.8 mm (5). The wall shows an attenuated echogenicity, and the serosa is visible as a hyperechoic linear structure (5). The contents can be a variable amount in normal conditions.

Laboratory rabbits are commonly used as an animal model of appendicitis in man (6, 7); however, spontaneous appendicitis was only described at *postmortem* in laboratory Japanese white rabbits (8) and *antemortem* in one pet rabbit with an acute abdomen (9). As per available literature, there is no therapeutic appendectomy and no bacterial pathogens were identified in the clinical case of appendicitis in rabbits. The aim of this article is to describe two spontaneous cases of appendicitis in pet rabbits, describe therapeutic appendectomy, and discuss microbial flora of the inflamed appendix.

## Case Presentation

### Clinical Case 1

A 5-month-old intact, female rabbit weighting 2,525 g was presented to the clinic because of restlessness followed by apathy, anorexia, teeth-grinding, and reduced faecal output. The clinical signs began the day before the rabbit was presented. Its diet included commercial pellets, vegetables, and *ad libitum* hay. Clinical examination revealed hyperactivity, normal body condition [BCS 3/5, (10)], skin tenting (estimated dehydration 5%), serous ocular discharge, purulent nasal discharge, severe discomfort on abdominal palpation, a palpable tubular abdominal structure in the mid-abdomen, and a rectal temperature of 41.1°C. Blood analyses showed a non-regenerative anaemia, monocytosis, hyperphosphatemia, hyperglobulinaemia, and hypercholesterolaemia. Urine analysis showed a pH of 6 and proteinuria. Radiography and abdominal ultrasound revealed a reduced amount of chymus in the stomach and intestines, and the presence of tubular structure with wall thickness of 4 mm with a rounded, closed end and a multilayered wall characteristic of the intestinal tract. These changes were suggestive of appendicitis. The client refused any further treatment, and the animal was euthanized. A *postmortem* examination revealed a thickened appendix with necrotic debris within its lumen. Histopathological examination of the appendix confirmed severe oedema of intestinal mucosa, multifocal to diffuse mixed, and neutrophilic inflammatory infiltrate, fibrinous exudate on the surface of mucosa and the presence of micro-abscesses within the necrotic mucosa. Aerobic and anaerobic cultivation showed the presence of pure growth of *Klebsiella variicola* sensitive to enrofloxacin, marbofloxacin, tetracycline, cefuroxime, trimethoprim sulphonamide, neomycin, and gentamicin and resistance to penicillin G, amoxicillin clavulanate, and bacitracin.

### Clinical Case 2

A 16-month-old neutered male rabbit weighting 1,380 g was presented to the clinic for a gradual decline in appetite and reduced faecal output over 3 days. It was fed on a commercial pelleted diet, vegetables, and hay *ad libitum* with no dietary changes. The rabbit had been examined 3 days previously by another practitioner because of mild apathy and reduced faecal output. At that time, blood sample results showed normal WBC, heteropenia, lymphocytosis, hyperglycaemia, and hyperproteinaemia (Table 1). The rabbit was treated with an oral analgesic drug meloxicam at 0.4 mg/kg q12h (Meloxoral 1.5 mg/ml, Le Vet B.V., Oudewater, Netherlands) and a prokinetic itopride at 10 mg/kg q12h (Itoprid, Pro Med, Hradec Kralove, Czech Republic). Syringe-feeding with a recovery diet for herbivores was advocated.

**Table 1:** Haematology, selected plasma chemistry parameters, and urine pH of a rabbit with appendicitis.

**Parameter**	**Clinical case 1**			**Clinical case 2**		
	**3 days before the presentation to the clinic**			**Day of the examination**	**10 days after the examination**	**Reference ranges (11, 12)**
Haematocrit	l/l	0.26	0.38	0.31	0.353	0.31–0.43
RBC	10^9^/l	4.25	6.01	4.98	5.82	4.5–6.9
Haemoglobin	g/l	87	128	109	117	110–144
MCH	pg	60.2	213	219	201	194–238
MCHC	g/l	340	344	347	331	323–345
MCV	fl	60.2	63.7	63.1	60.7	59.0–70.1
Platelets	10^6^/l	77	351	367	195	134–567
WBC	10^6^/l	6.52	4.94	13.53	6.67	4.1–10.8
Heterophils	10^6^/l	1.57	0.47	3.16	1.28	0.87–7.82
Heterophils	%	24.1	9.6	23.4	19.3	21–73
Lymphocytes	10^6^/l	2.59	2.87	7.43	4.79	0.36–6.58
Lymphocytes	%	39.7	58.1	54.9	71.8	9–64
Monocytes	10^6^/l	1.81	0.99	2.3	0.36	0.08–1.71
Monocytes	%	27.8	20	17	5.4	1–32
Eosinophils	10^6^/l	0.1	0.01	0.02	0.05	0.07–0.19
Eosinophils	%	1.5	0.2	0.1	0.7	0–0.7
Basophils	10^6^/l	0.45	0.6	0.62	0.19	0.06–1.1
Basophils	%	6.9	121	4.6	2.8	0–7
Total protein	g/l	71	83	76	68	61–77
Albumin	g/l	32	40	32	35	28–40
Globulin	g/l	39	43	44	33	21–37
Urea	mmol/l	8.4	6.5	7.9	8.2	3.6–8.6
Creatinine	μmol/L	144	79	89	122	71–159
Glucose	mmol/L	8.07	13.73	15.49	7.98	4.17–8.06
Phosphorus	mmol/L	2.77	1.43	1.48	1.32	0.39–1.58
Calcium	mmol/L	2.56	3.02	3.03	3.14	1.9–3.2
Urinalysis—pH		6	8	7	8	7.7–9.6

**Figure 1:**
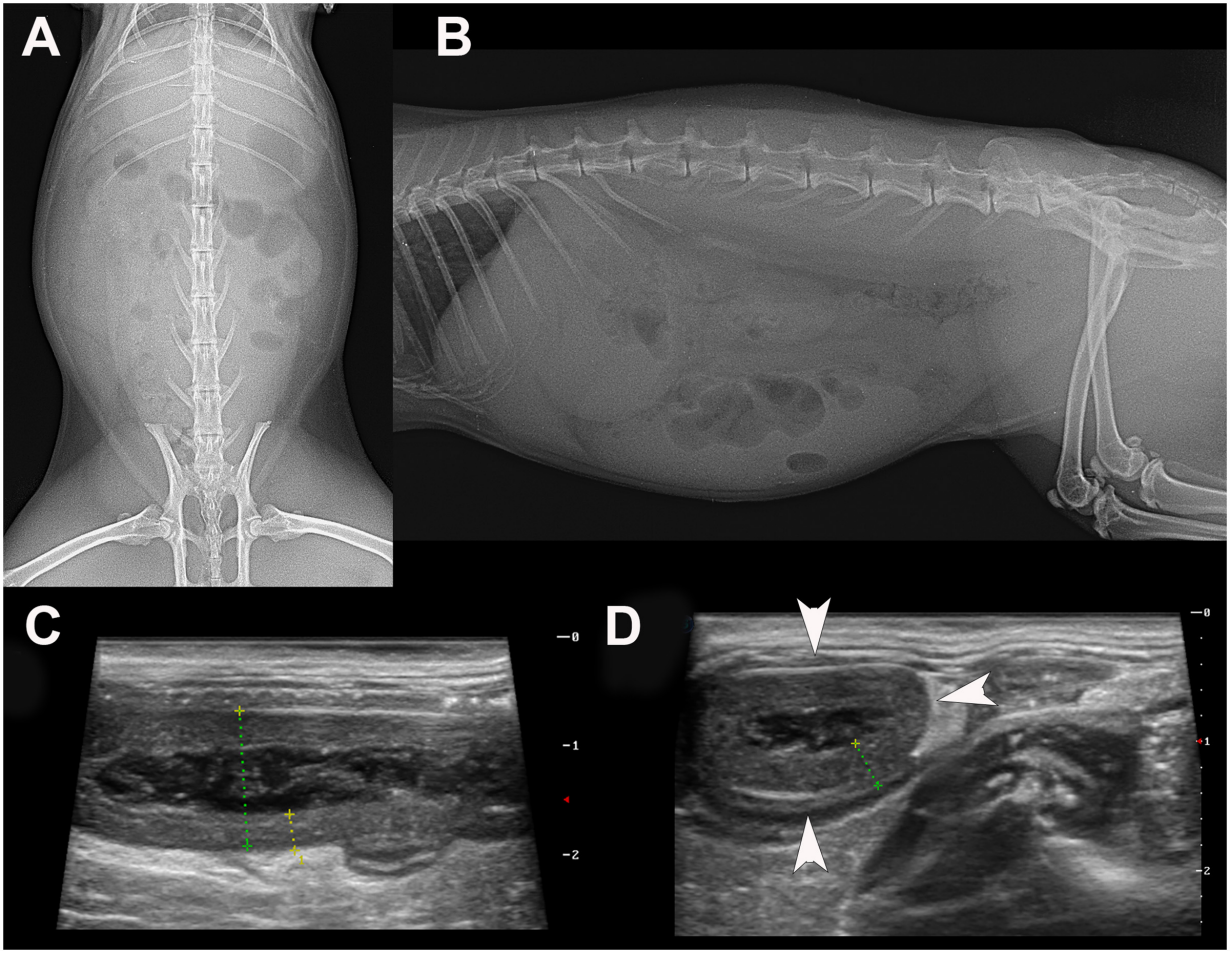
Ventrodorsal **(A)** and lateral **(B)** radiograph of the 16-month-old rabbit that was suffering from appendicitis. Note the soft tissue opacity due to chymus in the GI tract because the client had syringe-fed the animal with a recovery diet. A small amount of gas is seen in the caecum. The radiograph is inconclusive for the diagnosis of any GI tract disorder. On abdominal ultrasound, note the tubular organ on longitudinal **(C)** and transverse section **(D)**. Both views showed a thickened, hypoechoic wall with a lumen content of heterogeneous echogenicity, which was confirmed by surgery. The inflamed appendix was filled with necrotic material. The size of the whole of the transverse section of the appendix was 12.5 mm with a wall thickness of 3.7 mm.

Three days later, the rabbit was referred to the authors' clinic, where clinical examination revealed active to hyperactive behaviour, normal body condition (BCS 2.5/5), tachycardia, tachypnoea, skin tenting (estimated dehydration 5%), severe discomfort on abdominal palpation, a full stomach with a small amount of gas, a palpable tubular abdominal structure in the mid-abdomen, and a rectal temperature of 39.6°C. Blood analyses showed leucocytosis, lymphocytosis, monocytosis, hyperglycaemia, and hyperglobulinaemia (Table 1). Urine analyses showed pH of 7 (Table 1). Radiography and abdominal ultrasound revealed a reduced amount of chymus in the stomach and intestines (Figure 1). The tubular structure had a wall thickness of 4.2 mm with a rounded, closed end and a multilayered wall characteristic of the intestinal tract (Figure 1). These findings were suggestive of appendicitis.

Supportive treatment was started immediately and consisted of intravenous fluid therapy at 15 ml/kg/h (Ringerfundin, B. Braun, Melsungen, Germany) and a 5-ml bolus of 5% glucose, analgesic drug buprenorphine at 0.03 mg/kg i.m. (Bupredine Multidose, Le Vet Beheer B.V., Netherlands), a prokinetic drug metoclopramide at 1 mg/kg i.m. (Degan, Eurovet Animal Health, Bladel, Netherlands), an appetite stimulant pro toto at 2 ml (Catosal 10%, Bayer, Leverkusen, Germany), an antiulcer drug famotidine at 1 mg/kg i.m. (Quamatel, Gedeon Richter, Budapest, Hungary), a hepatoprotective drug at 0.1 ml/kg/pro toto (acidum methylphenoxypropionicum, Hepagen, Biopharm, Ždár, Czech Republic), and vitamin C at 30 mg/kg s.c. (acidum ascorbicum Biotika, Biotika Bohemia, Prague, Czech Republic). Meloxicam at 0.8 mg/kg s.c. (Meloxidolor 5 mg/ml, Le Vet Beheer B.V., Netherlands) was administered 4 h after the patient admission to the clinic, when it was stabilised with intravenous fluid therapy.

Anaesthesia was induced with medetomidine at 0.02 mg/kg i.m. (Domitor, Pfizer, Berlin, Germany) and ketamine at 3 mg/kg i.m. (Narkamon, Bioveta, Ivanovice na Hané, Czech Republic) followed by propofol at 0.3 ml to effect (propofol 1% MCT/LCT Fresenius, Fresenius Pharma Austria GmbH, Vienna, Austria). The rabbit was intubated using over the endoscope technique, and anaesthesia was maintained with isoflurane at 1.5–3% (Isoflurane Rhodia, Torrex Pharma GmbH, Wien, Austria). Continual intravenous fluid therapy with Ringerfundin was administered throughout the surgical procedure at a rate of 10 ml/kg/h. Perioperative monitoring consisted of observing respiration by close inspection of the thoracic wall, lung and heart auscultation, ECG monitoring, and pO_2_ (mean value of 99%) measurement using a pulse oximeter (Eickemeyer LifeVet® monitor, Eickemeyer, Tuttlingen, Germany) attached to the hind-limb fingers.

**Figure 2:**
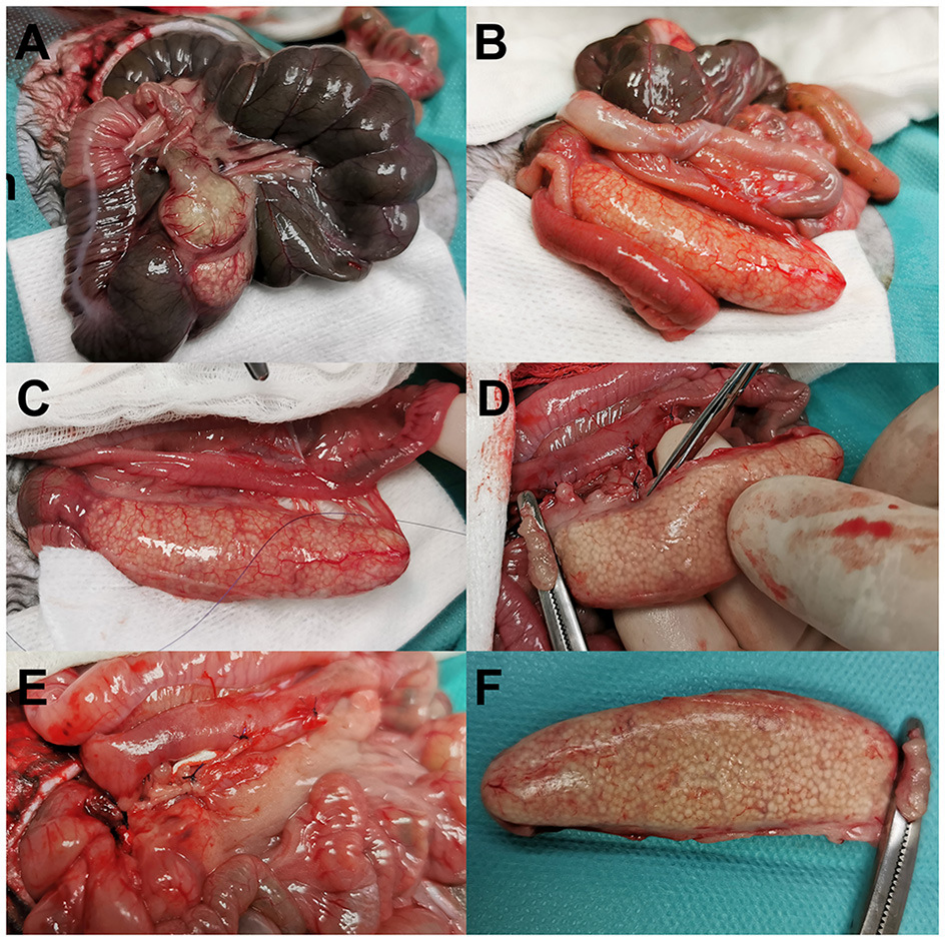
Intra-operative view of the 16-month-old rabbit with both a sacculitis and appendicitis **(A–F)**. Exploratory laparotomy showed a thickened wall of both the *sacculus rotundus* **(A)** and the vermiform appendix **(B)**. During appendectomy, all the vessels associated with the appendix were ligated **(C)** prior to excision of the appendix **(D)**. Postoperative view of a caecum after appendectomy **(E)** and excised vermiform appendix **(F)**.

A standard midline laparotomy confirmed a presence of small amount of free fluid in the abdomen (~10 ml) and a thickened *sacculus rotundus* and a thickened *processus vermiformis caeci* (appendix) with miliary yellowish lesions visible in the wall of these structures (Figure 2). All the vessels in the cecocolic fold were ligated (PDS 5-0, Johnson & Johnson Ethicon, Cincinnati, OH, USA), and the caecal body was transfixed with a suture and then transected to free the vermiform process (appendix). The appendix was resected, and the distal opening of the caecum was closed in two layers by an inverted suture pattern using polydioxanone PDS 5-0, Ethicon. The patency of the caecum was confirmed by gentle palpation of the suture between the thumb and index finger. Other perioperative findings included yellow colour of the mesenteric fat and mild splenomegaly. No signs of enteritis or pancreatitis were seen. After copious abdominal lavage with sterile saline warmed to 39°C, the abdominal wall was closed routinely using polyglactin 910 (Vicryl 4-0, Ethicon). The skin was stitched with a modified intradermal suture using polymer of glycolic acid (PGA Resorba 5-0, Resorba Medical GmbH, Nuremberg, Germany). Atipamezole at 0.15 mg/kg i.m. (Antisedan, Pfizer, Germany) was administered after surgery.

In the 4 h following surgery, the rabbit started to recover and did not show extreme pain on gentle abdominal palpation. Intravenous fluid therapy was continued, and analgesics (buprenorphine at 0.03 mg/kg i.m. q6h, and meloxicam at 0.8 mg/kg s.c. q12h) were given. To support intestinal motility, apart from metoclopramide, oral itopride at 10 mg/kg q12h (Itoprid, Pro Med, Czech Republic) and intravenous lidocaine at 100 μg/ml/h (Lidocaine Egis, EGIS Pharmaceuticals, Hungary) were also administered. The preoperative supportive treatment was continued for 6 days with fluid therapy. As antibiotics, a combination of marbofloxacin at 10 mg/kg i.m. q24h (Quiflox inj., Krka, Novo Mesto, Slovenia) and metronidazole at 20 mg/kg i.v. q12h (Entizol, Krka, Slovenia) was used. The rabbit was syringe-fed with recovery diet (EmerAid IC Herbivore, EmerAid, Cornell, IL, USA) and Supreme Science Recovery Plus, Supreme Petfoods, Ipswich, UK). It started to eat on its own and produce faecal pellets 2 days after surgery.

After 6 days, the patient was sent to home care with oral itopride at 10 mg/kg q12h, oral meloxicam at 0.8 mg/kg q12h, and oral marbofloxacin at 10 mg/kg q24h, for the next 10 days. Follow-up examinations at 10 days, at 1 month, and at 11 months after the surgery did not show any abnormal clinical or laboratory findings.

**Figure 3:**
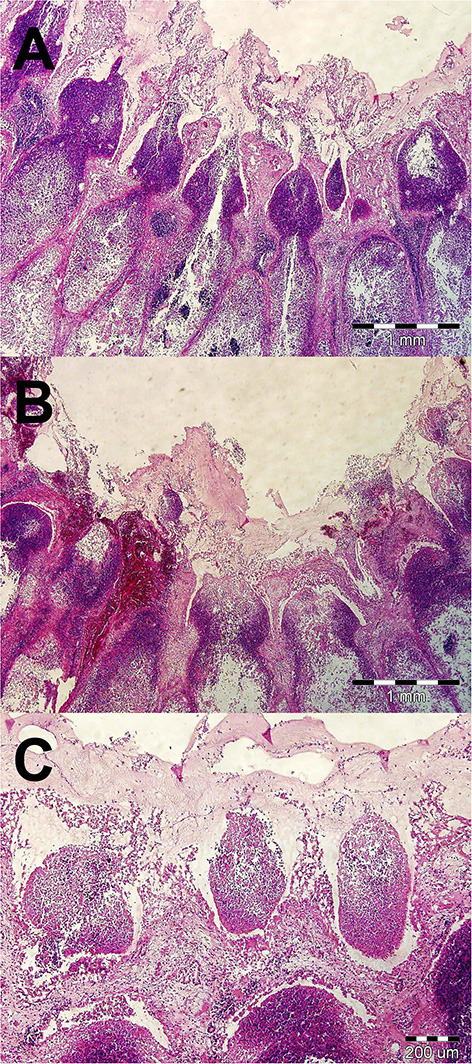
Histopathological examination of a vermiform process in a rabbit with appendicitis **(A–C)**. **(A)** Severe oedema of intestinal mucosa, multifocal to diffuse mixed and neutrophilic inflammatory infiltrate, and fibrinous exudate on the surface of mucosa. HE stain, ×40. **(B)** Areas of haemorrhages in mucosa and superficial fibrinous exudate. HE stain, ×40. **(C)** Formation of micro-abscesses in superficial part of mainly necrotic mucosa. HE stain, ×100.

Samples of the appendix wall and its necrotic contents were sent for histopathological and bacteriological testing. Histopathological examination of the appendix wall revealed the same findings as in the previous case (Figure 3). Aerobic culture and biochemical identification (MALDI-TOF Mass Spectrometry) (13) of the bacterial pathogens performed at the human-accredited laboratory identified the presence of pure culture of *Klebsiella variicola* sensitive to enrofloxacin, marbofloxacin, tetracycline, cefuroxime, trimethoprim sulphonamide, neomycin, and gentamicin and resistance to penicillin G, amoxicillin clavulanate, and bacitracin. *Bacteroides sp*. was isolated in low amounts and was sensitive to enrofloxacin, marbofloxacin, clindamycin, and metronidazole and resistant to tetracycline, trimethoprim sulphonamide, and penicillin G.

## Discussion

Although appendicitis in humans can be caused by direct luminal obstruction (faecolith, lymphoid hyperplasia, impacted stool or, rarely, by an appendiceal or caecal tumour), the full range of specific causes remains unknown. Recent theories focus on genetic factors, environmental influences, and infections (14).

In rabbits, the vermiform appendix differs from the vermiform appendix in man. It has a larger lumen, so the possibility of spontaneous obstruction is reduced. It also has a thinner muscularis and a different configuration of the mucosa and lymphoid tissue (15). No aetiology for spontaneous disease is described for appendicitis in rabbit, but, based on our observations, inflammation of lymphoid GI tissue associated with caecal dysbiosis was the probable cause in the two rabbits in this case report.

The most common aerobic organisms isolated from human appendicitis are *Escherichia coli, Klebsiella pneumoniae, Streptococcus* spp., *Enterococcus* spp., and *Pseudomonas aeruginosa* (16, 17). In the presented cases, *Klebsiella variicola* and *Bacteroides* sp. were the only isolates, which indicate severe aerobic dysbiosis. *K. variicola*, a member of Enterobacterales order, is a gram-negative, facultative anaerobic, non-spore-forming, non-motile rod-shaped bacteria. *K. variicola* is widely distributed among different groups of plants and has also been detected in rivers (18). *K. variicola* is considered as an emerging pathogen in humans causing sepsis, urinary tract, and GI tract infection (19). In animals, *Klebsiella* spp. are associated with infections of the urinary tract, respiratory tract, and sepsis (20). In rabbits, *K. pneumoniae* and *K. oxytoca* are described as a cause of necrotic and/or haemorrhagic enteritis and typhlitis (21, 22). In a normal rabbit caecum, the most common bacteria belong to phyla *Firmicutes* and *Bacteroides* (23). *K. variicola* can be one of the minority bacterial species found in rabbit intestine; however, at the time of writing, there are no published reports of this bacterium in the rabbit caecum. In the two rabbits described in this case report, the source of *K. variicola* remains unknown as both rabbits were on a different diet and were from different breeders. Fontana et al. (24) reported that *K. variicola* has been misidentified as *Klebsiella pneumoniae* due to its phylogenetic and biochemical properties to those of *K. pneumoniae*. It was estimated that 2.5–10% of *K. pneumoniae* isolates are an actual misidentification of *K. variicola*. Actually, *K. variicola* might be more frequent in the gut.

Antibiotic treatment (a combination marbofloxacin and metronidazole) was in the presented clinical case report aimed to prevent and treat aerobic and anaerobic infection. Antimicrobial sensitivity revealed sensitivity of *K. variicola* sensitive to enrofloxacin, marbofloxacin, tetracycline, cefuroxime, trimethoprim sulphonamide, neomycin, and gentamicin and resistant to penicillin G, amoxicillin clavulanate, and bacitracin. *Bacteroides* sp. was sensitive to enrofloxacin, marbofloxacin, clindamycin, and metronidazole and resistant to tetracycline, trimethoprim sulphonamide, and penicillin G. In a study of antimicrobial susceptibility of 100 *Klebsiella* animal clinical isolates, resistance was common against ampicillin (99%) and cephalexin (43%) but not against ceftazidime, ceftiofur, tetracycline, enrofloxacin, gentamicin, and trimethoprim–sulfamethoxazole (20). Authors were aware of using marbofloxacin as the antimicrobial drug of a second choice but were afraid of intramuscular or subcutaneous enrofloxacin administration, as it is associated with necrosis and haemorrhage in rabbits (25) and may negatively affect animal welfare. The authors strongly recommend following use of antimicrobial rules in animals (26). Use of oral administration of trimethoprim sulpha or tetracyclines may be another option.

The diagnostic and therapeutic approach to the acute rabbit abdomen should be the same as other emergency cases in rabbits (27, 28). GI stasis is one of the most common disorders of pet rabbits and is characterised by normal behaviour to apathy, abdominal discomfort, and gradual decline in appetite and production of small, dry faecal pellets (29, 30). In contrast, animals with gastric dilation (gastric bloat) suddenly become depressed and anorexic (31) and, with the onset of hypovolemic shock, body temperature declines. On clinical examination, both rabbits were described in this case report as restless, showed severe discomfort on abdominal palpation, and had elevated rectal temperature when they were presented to the clinic. Based on authors' observations, this behaviour with an elevated rectal temperature was associated with severe pain and inflammation of the appendix, as in humans (14).

Lymphocytosis in rabbits is associated with inflammatory changes of lymphoid organs, viral disease, lymphoproliferative disorder, and lead poisoning but is also seen with high adrenaline levels, which can be associated with increased activity and restlessness seen in the two presented rabbits (32).

Blood glucose measurements in clinical case 2 showed hyperglycaemia, which can be due to stress and pain. Harcourt-Brown and Harcourt-Brown (33) found that hyperglycaemia in anorectic rabbits (>20 mmol/l) was associated with conditions with a poor prognosis. The mean blood glucose in 15 rabbits with gastric dilation was 27.4 mmol/l, which was significantly higher than the mean blood glucose of 8.5 mmol/l of rabbits with GI stasis. Glucose concentrations can also increase to maintain serum osmolality in the face of sodium loss into the GI tract (34).

A diagnosis of appendicitis in humans is based on abdominal palpation, ultrasound examination (86% sensitivity), and computed tomography imaging (92.3% sensitivity) (14, 35). In the two presented cases, abdominal palpation and ultrasound examination were superior to abdominal radiography and clearly detected a thickened vermiform appendix. In rabbits, abdominal ultrasound can be problematic, especially when a gas is present in the intestinal tract, so thorough ultrasound examination is needed to evaluate the appendix.

Nicolletti et al. (5) described a negative correlation between age and appendix wall thickness, suggesting that lower values of thickness are associated with increasing age, caused by a physiological decrease in the immune function of the appendix in adult rabbits. In presented cases, appendix wall thicknesses were 4.2 and 3.7 mm, respectively.

There is only one published clinical case of appendicitis in a pet rabbit (9), and the rabbit was treated medically with non-steroidal anti-inflammatory drugs, prokinetics, and oral trimethoprim sulphate and metronidazole. Rabbits that were used as laboratory models of appendicitis were treated with intramuscular ceftriaxone and lincomycin daily for 5 days which decreased inflammation, but the rabbits were then euthanized (36).

Appendicitis in adults and children is a painful disease and is one of the most common conditions that require emergency abdominal surgery (35). In normal laboratory rabbits, experimental appendectomy has been performed successfully either by placing a ligature around the base of the appendix ~1 cm from the caecal body and transfixing it using 4–0 prolene, or by ligation of the appendiceal stump followed by a purse-string invagination of the caecal base. Appendectomy was performed using a LigaSure device placed ~1 cm from the transition appendix; appendiceal artery ligation has also been described (37). In the presented case of appendectomy, a conventional method of ligating the blood vessels in the cecocolic fold, transfixing the caecal body with a suture, and then transecting it before resecting the appendix was performed. The distal opening of the caecum was closed in two layers with an inverted suture pattern. This is the method that was described and used successfully by Souza et al. (37).

Spontaneous appendicitis classification and guidelines either for medical or surgical treatment of choice are not published in rabbits, but the authors recommend vermiform appendix excision because gangrenous appendicitis was diagnosed in both the presented cases. There was a high risk of subsequent GI tract perforation and death of the patient, especially in more advanced cases of appendicitis (15).

An important aspect of the appendicitis treatment is the pre- and postoperative multimodal supportive care. In the presented case, the rabbit after vermiform appendix excision recovered uneventfully. The rabbit survived 11 months to this date with no further GI tract disorders. Sacculitis cannot be treated using surgical excision, so supportive care and antibiotics are advocated.

In conclusion, restlessness or abnormally active behaviour associated with anorexia, abdominal pain, palpable abdominal mass, hyperglycaemia, lymphocytosis, and elevated rectal temperature may be indicative of a condition that causes inflammation and pain within the GI tract, such as appendicitis. Abdominal ultrasound is recommended in rabbits with GI tract disorders, as radiography can be inconclusive. Sacculitis and appendicitis should be included into the list of differential diagnoses for the rabbit acute abdomen and GI stasis syndrome. *K. variicola* may be associated with appendicitis in rabbits as a causative agent or in association with appendix intraluminal dysmicrobia.

## Data Availability Statement

The original contributions presented in the study are included in the article/supplementary materials, further inquiries can be directed to the corresponding author/s.

## Author Contributions

VJ was a major contributor in manuscript writing, analysing and interpreting the patient data, surgery, and manuscript writing. AP was a major contributor to the manuscript writing and literature review. ID, KH, and JC contributed to the manuscript writing and literature review. MS analysed and interpreted the patient data regarding histopathological diagnose. All authors have read and approved the final manuscript.

## Funding

This study was supported by the grant of the University of Veterinary Sciences Brno 2021ITA15.

## Conflict of Interest

The authors declare that the research was conducted in the absence of any commercial or financial relationships that could be construed as a potential conflict of interest.

## Publisher's Note

All claims expressed in this article are solely those of the authors and do not necessarily represent those of their affiliated organizations, or those of the publisher, the editors and the reviewers. Any product that may be evaluated in this article, or claim that may be made by its manufacturer, is not guaranteed or endorsed by the publisher.
